# Stay strong, stay healthy: exploring a predictive model of psychological adaptation among Macau students studying in Mainland China within the postcolonial context

**DOI:** 10.3389/fpsyg.2024.1346309

**Published:** 2024-04-17

**Authors:** Haiyan Feng, Li Zhang

**Affiliations:** ^1^School of Journalism and Communication, Xiamen University, Xiamen, China; ^2^School of Journalism and Communication, Tsinghua University, Beijing, China

**Keywords:** psychological adaptation, postcolonialism, language proficiency, identity, intergroup contact, quality of contact, Macau, Mainland China

## Abstract

The prevailing research on adaptation primarily centers around the settlement of international students and immigrants in different cultural environments. However, there is a notable gap in understanding the intra-cultural adaptation process for individuals from postcolonial areas when relocating to their home country. The primary focus of the current study lies in constructing a predictive model that delineates the psychological adaptation experienced by Macau students studying in Mainland China. In total, two hundred and fifty-five Macau students completed a questionnaire which assessed variables falling into two categories: identity-related variables, such as language proficiency and identity, and intergroup-related variables, including intergroup contact and the quality of contact, and psychological adaptation. The findings from the present study revealed that identity and quality of contact continued to make significant contributions to psychological adaptation in intra-cultural environments as in inter-cultural environments, whereas language proficiency and intergroup contact were unrelated to psychological adaptation in intra-cultural adaptation. The present study extended the adaptation research by transporting hypotheses and findings from inter-cultural adaptation and testing their validity and applicability in postcolonial contexts. The findings also provided practical implications for Chinese education institutions and policy-makers.

## Introduction

The existing literature dedicated to understanding the psychological adaptation of international students consistently highlights the significance of language, identity, and intergroup contact in facilitating the integration of sojourners into a new cultural environment (e.g., Safdar et al., 2003; English and Zhang, 2020; Sheng et al., 2022). However, the aforementioned list of predictors in acculturation is particularly relevant to individuals who come from a vastly distinct cultural background. Surprisingly, limited attention has been given to studies focusing on the psychological adaptation of students from previously colonized areas who relocate to their home country. Although there are many cultural similarities between the colonized areas and their motherland, the disparities in language, educational system, political structure, and other issues stemming from colonization have presented formidable obstacles to the psychological adaptation of students who come from colonial enclaves and study in home country (Li and Bray, 2007; Xu, 2019; Lee et al., 2020). In fact, for these sojourners, adjusting to the life in motherland has been proven more challenging than adjusting to a distinct culture since the motherland is “more foreign than a foreign country” (Yu and Zhang, 2016).

Among all the former colonized areas, Macau, a former Portuguese colony, has stood out as a relatively successful example in reincorporating to the motherland. Especially compared with its eye-catching counterpart Hong Kong, a former British colony where there has been ongoing tension and conflict with mainland Chinese and the ruling Chinese government regarding issues of governance, autonomy, and civil liberties after the return, Macau is undoubtedly a more successful example in decolonization and reincorporation. Against this social background, there has been a massive outflow of Macau students to higher education institutions in Mainland China. According to the Chinese Ministry of Education, Mainland China has become the most favored destination for Macau students to pursue higher education since 2012 (Zou, 2022). The 2022 Hong Kong, Macau, and Taiwan Joint Recruitment Tests (HMTJT) also saw 21% more Macau applicants than in the previous year (Sohu News, 2022).

The current study, therefore, seeked to construct a predictive model delineating the psychological adaptation of Macau students into Mainland China in the postcolonial context. Specifically, grounded in the field of cross-cultural psychology (Berry et al., 2006), this study investigated two categories of variables, i.e., identity-related variables and intergroup-related variables, and their connections with psychological adaptation and respective contributions to the adaptation process. We believe this research contributes to the current literature in three ways. First, as advocated by Berry and colleagues (Berry and Dasen, 1974; Berry et al., 1999), the first and most obvious goal of cross-cultural psychology is to transport hypotheses and findings to other cultural contexts in order to test their validity and applicability in other cultures. Therefore, the current study responded to these calls by adding the unique Chinese postcolonial context to the cross-cultural psychology literature. Moreover, since Macau offers a rare example where there is a continuous and massive outflow of students to higher education institutions in Mainland China, this exploratory study provides an overall picture of significant adaptation stressors or challenges that students from former colonies might encounter when adapting themselves to home country. In this way, the present study broadened the scope of postcolonial research into the unique Chinese context. More importantly, relevant recommendations for university support and policymakers to facilitate the adaptation of Macau students studying in Mainland China were discussed.

## Literature review

### Language proficiency, identity, and psychological adaptations in inter-cultural adaptation

Psychological adaptation refers to a subjective sense of enduring life satisfaction (Sharma and Sharma, 2010); it is crucial for a positive cross-cultural experience during international expatriation and sojourning (Huff et al., 2014; Chu and Zhu, 2023). Individuals undergo psychological adaptations when confronted with a shifting cultural environment (Ye and Dong, 2021). Sojourners who undergo more favorable psychological outcomes, such as reduced depression, diminished loneliness, lower anxiety, and decreased stress, demonstrate a higher level of adaptation to the new cultural environment (Black and Stephens, 1989). As argued by Ward and Kennedy (1994), cross-cultural adjustment is determined by both psychological adaptation and socio-cultural adaptation. Consequently, a large body of inter-cultural adaptation literature was dedicated to uncovering the antecedents of successful psychological adaptation in inter-cultural environments (e.g., Martínez García et al., 2002; Chae and Foley, 2010; Jibeen and Khalid, 2010; Mähönen and Jasinskaja-Lahti, 2013; Park et al., 2014).

Studies have consistently demonstrated that language and identity are strong predictors of psychological adaptation in inter-cultural adaptation (e.g., Berry et al., 2006; Lou and Noels, 2020). Proficiency in language is a crucial skill for comprehending and engaging with individuals in social, academic, and professional settings. This proficiency directly influences people’s psychological adjustment within these contexts (Brunsting et al., 2018; Luo et al., 2019). Identity, too, is recognized as a crucial resource on which the psychological adaptation of minority individuals is based (e.g., Phinney, 1996; Vedder and Virta, 2005). Prior studies have revealed that language learning and identity development co-evolve over time (Bourhis et al., 1973; Fishman and García, 2010). Therefore, the present study views language and identity as one group of variables.

Scholars have proved language proficiency to be an antecedent of successful cultural adaptation (Clément, 1986; Noels et al., 1996). Hence, this study posited that language proficiency will relate positively to psychological adaptation in postcolonial circumstances. Notably, there are conflicting findings as to how identity influences psychological adaptation among ethnic minorities. Certain studies indicate that stronger identification with one’s ethnic group correlates with better psychological adjustment, as evidenced by findings from Berry et al. (2006) and Phinney et al. (2001). However, Lou’s empirical study located in the postcolonial context observed that a close identification with China led to better cultural adaptation and greater acceptance of other groups when Macau students interact in Mainland China (2021). This finding is consistent with a study of Hong Kong students studying in Mainland China, which showed that a local Hong Kong identity (vs. a Chinese national identity) was associated with worse adaptation experiences (Yu et al., 2023). Based on the existing literature, the current study hypothesized that Macau students with a strong national identity (i.e., Chinese), instead of a local identity (i.e., Macanese), will have better psychological states.

### Intergroup contact, quality of contact, and psychological adaptations in inter-cultural adaptation

The importance of intergroup contact is also underscored in the context of cross-cultural transitions. Prior investigations have offered empirical evidence supporting the significance of intergroup contact in facilitating psychological adjustment., and demonstrated that the more sojourners interact with hosts, the more likely they will have better psychological states (e.g., Searle and Ward, 1990; Liebkind, 1996; Ward and Masgoret, 2004; Te Lindert et al., 2022). In fact, intergroup relations between hosts and sojourners were believed to be the most influential factors associated with adaptation results, i.e., social support, social connectedness, and reduced prejudice (Lin, 2008; Cao et al., 2018). Therefore, this study also investigated whether intergroup contact continues to make the most significant contribution to psychological states in the postcolonial context when all predictors are introduced at the same time.

Furthermore, studies have found that sojourners who engage in more satisfying and positive interactions, characterized by pleasantness, intimacy, and cooperation, with host nationals tend to experience better emotional states (Tropp, 2003; Berry et al., 2006). This is consistent with Allport’s intergroup contact hypothesis which speculated that intergroup contact would maximally reduce prejudice when four optimal conditions were met, i.e., equal status between the groups, common goals, intergroup cooperation, and the support of the authorities, law, or custom (Allport, 1954). However, Pettigrew and Tropp (2005), a leading authority on intergroup attitudes, indicated that while the proposed conditions enhancing the quality of intergroup contact are important, they are not necessary for achieving positive effects. Therefore, it is necessary to investigate whether quality of contact will facilitate better psychological outcomes when intergroup contact is introduced at the same time. As a matter of fact, the argument that positive social contact with acquaintances and even strangers is associated with higher levels of happiness has been confirmed by some cross-cultural studies (e.g., Holt-Lunstad et al., 2010; Van Lange and Columbus, 2021). Based on the above literature, it was assumed in this study that quality of contact also positively correlates with psychological adaptation.

### Intra-cultural adaptation in the Macau postcolonial context

Scholars in postcolonial studies contend that individuals who have experienced postcolonial situations represent unique research subjects. They differ not only from locals in their home countries due to distinct languages, political systems, and educational practices shaped by postcolonial experiences but also from “pure” internationals, given the cultural similarities between previously colonized regions and their motherland (Fishman and García, 2010; Yan, 2017). In parallel with the postcolonial research, the inter-cultural adaptation research also argued that, like sojourners adapting to a distinct culture, people adapting to their home country must mitigate language barriers, tackle identity conflict, consolidate values, and continue to improve inter-cultural competence (Chou, 2010; Lee et al., 2020). As an illustration, Lou (2021) carried out a quantitative study revealing a positive association between Chinese national identity and Mandarin language proficiency (the common language of Mainland China) with the cultural adaptation of Macau students studying in Mainland China. Nevertheless, apart from Lou’s study, there has been a limited amount of research exploring the diverse factors influencing psychological adaptation among educational sojourners making the transition to their home country from previously colonized regions. In the following, this study discusses the context of Macau in relation to Mainland China to better understand the necessity of adding postcolonial research subjects to the adaptation research.

Macau is a Special Administrative Region of China where the political formula of “One Country, Two Systems” has been implemented since 1999, after more than 400 hundred years of occupation by Portugal (Cheng, 1999). Consequently, the educational, political, economic, and legal systems of Macau differ from those of Mainland China (Lee et al., 2020). After Macau’s reunification with China, many of its distinct systems have endured, as Macau retains a degree of autonomy from Mainland China. Among all the differences, language and identity are key agenda items for decolonization and renationalization by the Chinese government (Chou, 2010; Yan, 2016).

The complexity in language is one of distinguishing features in Macau society. Having once been a Portuguese colony, Macau stands out as the sole region in China where Cantonese, a Chinese dialect, and English, a global language, hold *de facto* official statuses. Mandarin also holds a quasi-official status, while Portuguese remains another official language. However, Cantonese is the most widely used language for social communication in various domains, such as home, work, education, and media (Hok-Shing and Chan, 2015). This is because Macau was originally a part of Guangdong, one of China’s southernmost provinces where Cantonese is the native tongue. Since the handover, the percentage for Mandarin as the usual language has increased from 1.2% (1999) to 1.6% (2001), 3.2% (2006), and 4.7% (2021) (The Documentation and Information Centre of the Statistics and Census Service, 2021). However, Cantonese remains the most important language since 81.0% Macanese use Cantonese in daily communication as well as in Legislative Council meetings and on official occasions (Yan, 2016).

For Macau students studying in Mainland China, adjusting to the language presents a significant challenge because Mandarin and Cantonese differ greatly not only in pronunciation but also in Romanization, characters, vocabulary, and grammar (Lee et al., 1996). Mandarin is the common language in mainland China, whereas Cantonese remains the most used language in Macau. For one, Mandarin and Cantonese vary in pronunciation due to discrepancies in phonetics and tonal aspects. For another, Mandarin uses Hanyu Pinyin for transliteration while Cantonese romanization system uses Jyutping for transliteration. More importantly, simplified character (also known as Simplified Chinese) is used for writing almost exclusively everywhere in Mandarin-speaking Mainland China while traditional character (also known as Traditional Chinese) is used prevalently for writing in Cantonese-speaking Macau. In a word, Mandarin and Cantonese are not mutually intelligible due to the abovementioned differences.

Another notable characteristic of Macau society is the complexity surrounding identity. In common with other postcolonial settings, identity serves as a battleground for political contention, where diverse discourses intersect, embodying the reclaiming of political traditions and legacies. In Macau, the post-handover identity comprises the local, the national and the international components (Lam, 2010). The newly formed postcolonial regime prioritizes the reconstruction of a cohesive Chinese national identity. This endeavor aims to foster a collective sense of shared Chinese history, memories, symbols, and cultures, along with a belief in the significance of governance practices conducive to societal well-being. Simultaneously, it’s important to acknowledge that the Macau government not only acknowledges the existing local identity, which comprises a captivating blend of colonial, historical, and indigenous characteristics, but also proactively reshapes an initially fragile local identity into a robust one (Ho, 2011). What’s remarkable is that the key aspect of local identities is not merely whether individuals were born in the city, but rather their experience of receiving primary education in Macau. These “local persons” are accustomed to the liberties of religion and expression, a certain degree of cosmopolitanism, and crucially, proficiency in Cantonese. Ideally, in the formation of a Chinese national identity, local identities are either given secondary significance or integrated into the overarching identity (Lou, 2021). However, conflicts between a national Chinese identification and a local identity always exist in Macau. Occasionally, the prevailing local identities have posed a clear threat to the national reintegration of Macau (Kaeding, 2010). For example, Chou’s (2010) study found that the identity of Macau residents is primarily centered on the local culture of the city, with a comparatively lower sense of belonging to the national identity.

### The present study

One crucial objective of adaptation research is to transfer hypotheses and findings to different cultural contexts to assess their validity and applicability (Berry and Dasen, 1974; Berry et al., 1999). The current research aims to answer such call by constructing a predictive-model of psychological adaptation experienced by sojourners from previously colonized areas (i.e., Macau) transitioning to their motherland (i.e., Mainland China). To fulfill this goal, the findings from adaptation studies concerning the predictors of psychological adaptation ware applied to the postcolonial setting of Macau. Drawing upon the reviewed literature, we formulate the following research questions and hypotheses:

*RQ1:* What are the relationships between identity-related variables and psychological adaptation in the Macau postcolonial context?

*H1a:* A positive correlation is hypothesized between language proficiency and psychological adaptation.

*H1b:* A positive correlation is hypothesized between national identity and psychological adaptation.

*RQ2:* What are the relationships between intergroup-related variables and psychological adaptation in the Macau postcolonial context?

*H2a:* A positive correlation is hypothesized between intergroup contact and psychological adaptation.

*H2b:* A positive correlation is hypothesized between the quality of contact and psychological adaptation.

*RQ3:* Does intergroup contact continue to make the most contribution to psychological adaptation in the Macau postcolonial context?

*H3:* Intergroup contact makes the most contribution to psychological adaptation when all the predictors are considered.

## Methods

### Participants and procedure

The participants were enlisted using convenience sampling, a method that is a frequently used in cross-cultural adaptation studies involving international students (e.g., Zhou et al., 2008; Brisset et al., 2010; An and Chiang, 2015). Situated in the Chinese postcolonial context, the existing literature mostly adopted a qualitative approach. For example, Yu and Zhang (2016) conducted focus group discussions to explore Mainland students’ adaptation to life and study in Hong Kong. Zhang (2019) conducted in-depth interviews to examine the mainland Chinese students’ complex sociolinguistic experiences in Macau. In one exception, Dai and Liu (2021) used convenience methods that included 63 mainland college students studying in Hong Kong and Macau to investigate their adaptation levels. In another line of study that explores the adaptation of students of former colonial enclaves studying in Mainland China, a dominant literature also employed qualitative methods (e.g., Lee et al., 2020; Yu et al., 2023). In one exception, Lou (2021) adopted a quantitative approach to examine the importance of language and social identity of Macau students transitioning to universities in Mainland China. He used the convenience sampling method and recruited 102 participants from three mainland Chinese universities. The existing literature has showcased that the subjects involved (i.e., Macanese students studying in mainland Chinese whose first language is Cantonese) are highly underrepresented and hard to reach. Therefore, the current study adopted convenience sampling methods to recruit participants.

In the first step, the study was first approved by the Office of Taiwan, Hongkong and Macau Affairs at two Chinese key universities, one located in northern China, and the other in southern China. There are three reasons for such a selection. The two universities housed most Macau students (the northern university ranked 2nd and the southern university ranked 6th) according to a reported released by Ministry of Education of the People’s Republic of China.^1^ Second, the geographical environment and dietary habits are substantially different between northern and southern regions in China (Song and Cho, 2017).^2^ For example, rice is a main staple food in the southern region, while wheat is a main staple food in the north. Third, alongside the geographical and dietary differences, there are also economic, cultural, and linguistic differences between northern China and southern China. For example, studies have shown that those from the southern regions are more likely to be cooperative, interdependent, and group-oriented (Ma et al., 2016). Considering the distinguishable regional differences between northern and southern China, the current study included both a northern university and a southern university to control the influence of “region” in Macau students’ psychological adaptation. It’s noteworthy that, regardless of the substantial differences between southern China and northern China, both regions are Mandarin speaking.

In the second step, the participants were recruited through the two offices based on their enrolled Macau student list. Interested participants emailed the researchers and received an electronic copy of the consent form and questionnaire. Participants provided consent, filled out the questionnaire, and received a 10 RMB gift upon completion. The questionnaire was administered in Traditional Chinese, which is the participants’ first language. The data were collected in November 2022. Participation in the study was entirely voluntary, and the confidentiality of participants was strictly upheld. The research included 255 participants who were Macau-born students, and Cantonese served as their first language.

### Measures

#### Language proficiency

Participants were requested to assess their proficiency in speaking, listening, reading, and writing of Mandarin Chinese using a 5-point scale (1 = very poor, 5 = excellent). Previous studies have identified a strong to moderate correlation between self-assessed language proficiency and the objectively measured language proficiency (Li and Zhang, 2021). The measure demonstrated good reliability (Cronbach’s alpha = 0.845).

#### Identity

A hierarchical identity framework, distinguishing between an inclusive national identity and an exclusive local identity, is commonly utilized in research investigating identity in Macau and Hong Kong. Studies, including those conducted by Brewer (1999), Fu et al. (1999), and Lou (2021), have shown good reliability and validity, supporting the continuum of this measure. Therefore, in the current study, participants were requested to select the option that best characterized their identity from the following choices: 1 = Macanese; 2 = Macanese primarily and Chinese secondarily; 3 = Chinese primarily and Macanese secondarily; and 4 = Chinese. Higher scores suggested a stronger identification with the Chinese identity.

#### Intergroup contact

Intergroup contact in this study referred to online contact between Macau students and the mainland Chinese because face-to-face contact was frequently disrupted when the questionnaire was collected due to the draconian control measures under the zero-Covid policy. To evaluate online contact, three items adapted from prior studies were employed. The items included: “In the past week, the number of days you contacted with mainland Chinese via social networking sites (QQ, WeChat, etc.),” “On an average weekday, how long do you communicate with mainland Chinese via social networking sites?,” and “On an average weekend, how long do you communicate with mainland Chinese via social networking sites?” (Bonetti et al., 2010). For the first item, responses were coded as follows: 1 = none, 2 = 1–2 days, 3 = 3–4 days, 4 = 5–6 days, and 5 = every day. Regarding the latter two items, responses were as follows: 1 = less than 15 min, 2 = between 15 min and 1 h, 3 = 1–2 h, 4 = 3–4 h, and 5 = more than 4 h. The measure demonstrated good reliability (Cronbach’s alpha = 0.886).

#### Quality of contact

The variable of quality of contact was assessed through six specific contact conditions, each rated on a 6-point bipolar scale: superficial–intimate, involuntary–voluntary, equal status–unequal status, cooperative–competitive, accepting-rejecting, and unpleasant–pleasant. Higher scores indicated a more positive contact experience with mainland Chinese. The reliability of the measure was found to be good (Cronbach’s alpha = 0.868).

#### Psychological adaptation

Past research often used indicators of life satisfaction to assess psychological adaptation (e.g., Sharma and Sharma, 2010; Best et al., 2014). The scale was evaluated using five questions adapted from Diener et al. (1985). The items comprise statements such as “The conditions of my college life are excellent,” “In most ways, my college life is close to my ideal,” “So far, I have gotten the important things I want in college life,” “I am satisfied with my college life,” and “If I could live my college life over, I would change almost nothing.” The response choices ranged across 5-point scales from 1 = strongly disagree to 5 = strongly agree. The measure exhibited strong reliability with a Cronbach’s alpha of 0.874.

#### Control variables

Given that demographic factors such as age, gender, education level, region, and length of residence in Mainland China have consistently emerged as predictors in acculturation studies within the Chinese context (e.g., Jiang and Shypenka, 2018; Xu, 2019; Lou, 2021; Sheng et al., 2022), they were incorporated as control variables in this study. For age, participants were asked to fill in their ages. For gender, participants were asked to indicate their gender, with 1 for male and 0 for female. For region, participants were asked to specify the region where they were studying, with 1 for southern China and 0 for northern China. Participants were required to select their education level from the following options: 1 = freshman, 2 = sophomore, 3 = junior, 4 = senior, or 5 = graduate. Participants were also requested to provide the length of their residency in Mainland China.

### Statistical analyses

In this section, two subsections were undertaken. First, the descriptive information and correlation matrix of the main variables were computed. Second, a predictive model of psychological adaptation was constructed using the hierarchical multiple regression analysis.

## Results

### Preliminary analysis

Descriptive analysis shows that the average age of these participants was 20.66 years (SD = 1.97, range = 17–30 years). The sample consisted of more females (73.3%) than males (26.7%). The distribution of education levels among the participants was as follows: freshman (12.5%), sophomore (23.5%), junior (17.6%), senior (30.6%), and graduate (15.7%). Out of all the participants, 147 (57.6%) were enrolled in a university in southern China, while 108 (42.4%) were studying in a university in northern China. On average, the participants had been living in Mainland China for 4.07 years, with a range of 0.6 to 10 years.

Table 1 displays the means, standard deviations, and Pearson correlations of the main variables. The findings indicate a positive correlation between language proficiency and national identity, intergroup contact, quality of contact, and psychological adaptation. Moreover, national identity exhibited a positive correlation with intergroup contact, quality of contact, and psychological adaptation. Intergroup contact demonstrated a significant correlation with the quality of contact and psychological adaptation, while the quality of contact was positively correlated with psychological adaptation.

**Table 1:** Means, standard deviations, and Pearson correlations of the main variables.

Variables	1	2	3	4	5
1. Language proficiency	1				
2. Identity	0.258^**^	1			
3. Intergroup contact	0.244^**^	0.281^**^	1		
4. Quality of contact	0.355^**^	0.223^**^	0.351^**^	1	
5. Psychological adaptation	0.199^**^	0.368^**^	0.240^**^	0.340^**^	1
*M*	4.018	2.61	2.417	3.943	2.871
*SD*	0.689	0.875	0.889	0.733	0.88

*N* = 255. ^**^p < 0.01.

### Regression analysis

Prior to the hierarchical multiple regression analysis, we assessed the potential for multicollinearity by examining correlation indices. The findings indicated that the inter-correlations among the independent variables remained below the recommended threshold of 0.6, and the tolerance scores for each variable exceeded 0.5. Therefore, a predictive model was formulated, and the predictors were introduced in three distinct blocks, with psychological adaptation serving as the dependent measure. First, the control variables were included to control for potential influences on psychological adaptation (Step 1). Second, identity-related variables were introduced in Step 2, followed by the addition of intergroup-related variables in Step 3. The results of this predictive model can be observed in Table 2.

**Table 2:** Hierarchical multiple regression model predicting psychological adaptation.

Predictors	Step 1	Step 2	Step 3
Demographic variables			
Age	0.251^**^	0.208^*^	0.228^**^
Gender	0.034	0.035	0.027
Education level	−0.270^**^	−0.177	−0.167
Region	0.221^***^	0.171^**^	0.172^**^
Length of residence	0.196^**^	0.048	0.018
Identity-related variables			
Language proficiency		0.089	0.002
Identity		0.273^***^	0.227^***^
Intergroup-related variables			
Intergroup contact			0.077
Quality of contact			0.246^***^
Adjusted *R^2^*	0.108	0.176	0.237
*R^2^*	0.126	0.199	0.264
*R^2^ change*	0.126^***^	0.073^***^	0.065^***^

*N* = 255. Gender 1 = male, 0 = female; Region 1 = southern China, 0 = northern China; Cell entries are standardized beta weights for each step. ^***^p < 0.001; ^**^p < 0.01; ^*^p < 0.05.

The overall model demonstrated a significant prediction of the psychological adaptation levels of Macau sojourners studying in Mainland China (*F*_(3, 245)_ = 9.755, *p* < 0.001), explaining 26.4% of the total variance. The findings revealed that the identity-related variables explained 19.9% of the variance in psychological adaptation (*R^2^*_change_ = 0.073, *F_change_* (2, 247) = 11.251, *p* < 0.001). A further 6.5% of the variance in psychological adaptation was explained with the addition of intergroup-related variables (*R^2^*_change_ = 0.065, *F_change_* (2, 245) = 10.793, *p* < 0.001). When all variables were introduced, the main variables of identity and quality of contact emerged as significant predictors of psychological adaptation.

Regarding demographic variables, age and region were positively correlated with psychological adaptation in all the four regression models. That means the older the participants are, the more likely they will experience better psychological states in Mainland China. In addition, Macau students who studied at a university in southern China experience better psychological states than those who studied at a university in northern China. This aligns with previous studies suggesting that students tend to adapt more effectively to cities with cultural and climatic similarities (e.g., English et al., 2019). Macau and southern China bear more similarities than Macau and northern China. First, Macau and southern China are geographical similar, where the climate is both sub-tropical and monsoonal. Both regions have long summers that are hot and humid, whereas northern China have long winters that are cold and dry. Second, Macau and southern China share similarities in dietary habits. For example, rice is a main staple food in both areas, while wheat is a main staple food in northern China. Moreover, Macau and southern China share substantial cultural similarities including worshiping Earth God and Kitchen God (Chinese domestic gods recognized in Chinese folk religion and Taoism), practicing Feng Shui (a Chinese geomancy which claims to use energy forces to harmonize individuals with their surrounding environment), and celebrating Winter Solstice Festival, to name a few, whereas such customs and celebrations are largely absent in northern China.

Significantly, gender did not exhibit a significant association with psychological adaptation in any of the models. Finally, when only the control variables were included in the regression, a negative correlation was observed between education level and psychological adaptation, while a positive correlation was found between the length of residence and psychological adaptation.

## Discussion

The main objective of this study was to extend hypotheses and findings from intercultural adaptation research to a postcolonial context, specifically by developing a predictive model for the psychological adaptation of Macau sojourners transitioning to Mainland China. The hypotheses regarding the prediction of psychological adaptation were largely supported by the findings.

With reference to RQ1, two hypotheses were tested. According to the correlations model of the main variables (Table 1), perceived language proficiency was positively associated with psychological well-being (*b* = 0.199, *p* < 0.01). However, according to the hierarchical multiple regression model (Table 2), language proficiency became unrelated to psychological adaptation. Hence, an additional regression model was executed to delve deeper into the predictive power of language proficiency. In this model, language proficiency was introduced into the regression with demographic variables before (as opposed to after) identity. The finding from this supplementary analysis indicated that language proficiency was significant prior to the introduction of identity into the regression model (*b* = 0.121, *p* < 0.01). However, when identity was entered, language proficiency became insignificant (*b* = 0.089, *p* = 0.091). Upon the inclusion of identity into the model, language proficiency lost its significance and predictive influence on the psychological adaptation of Macau sojourners. Therefore, H1a was not supported. This seemed to conflict the previous finding that Macau students who were more proficient in Mandarin adjusted better in mainland settings and had a lower level of depression (Lou, 2021). However, if we consider the results of correlational analysis and supplementary analysis altogether, language proficiency might be supportive of other factors that feed more directly into psychological adaptation. Therefore, future research could explore the possibility of a mediating or moderating role of language proficiency underlying the mechanisms between identity, quality of contact, and psychological adaptation.

Consistent with our expectations, identity is a strong and consistent predictor of psychological adaptation in the postcolonial context. In other words, the more the Macau sojourners identify with a national identity (i.e., Chinese) over a local identity (i.e., Macanese), the more likely they will experience better psychological states. The adaptation literature presents conflicting findings regarding whether a national identity or a local/ethnic identity leads to better intercultural adaptation results (Phinney et al., 2001; Berry et al., 2006; Lou, 2021). This study confirmed that a national identity, instead of a local identity, helps sojourners adapt better when relocating to their motherland in the postcolonial context. Hence, H1b was supported.

With reference to RQ 2, two hypotheses were examined. Contrary to our expectations, intergroup contact was unrelated to psychological adaptation in the postcolonial context. Therefore, H2a was not supported. In contrast, quality of contact remained a strong and consistent predictor of psychological adaptation in the postcolonial context. That means, Macau students who have more pleasant and satisfying contact with the mainland Chinese will experience better emotional states. Hence, H2b was supported.

Regarding RQ3, an additional regression model was executed to delve deeper into the predictive power of intergroup contact. In this model, intergroup contact was introduced into the regression before (as opposed to after) variables of quality of contact, language proficiency, and national identity. The findings from this supplementary analysis indicated that intergroup contact was significant prior to the introduction of other variables into the regression model (*b* = 0.204, *p* < 0.001). However, when quality of contact was entered, intergroup contact became marginally significant (*b* = 0.118, *p* = 0.058). Upon the inclusion of language proficiency and national identity into the model, intergroup contact lost its significance and predictive influence on the psychological adaptation of Macau sojourners. Therefore, H3 was not supported.

Notably, quality of contact remained significant in all regressions (*b* = 0.266, *p* < 0.001; *b* = 0.260, *p* < 0.001; *b* = 0.246, *p* < 0.001). This finding suggested that, although intergroup contact proves to be the most influential and consistent factor associated with adaptation results in inter-cultural environments (Lin, 2008; Cao et al., 2018), it is quality of contact, rather than intergroup contact, that makes significant contribution to psychological states in intra-cultural environments. The findings are consistent with Allport’s intergroup contact hypothesis that held optimal factors facilitating high quality of contact are essential conditions for intergroup relations to yield positive results. However, the existing adaptation studies have focused more on the valence of intergroup contact rather than the optimal conditions outlined by Allport. Few studies have explored the effects of both intergroup contact and the quality of contact on cross-cultural adaptation simultaneously. Our study included both intergroup contact and the quality of contact at the same time and indicated that quality of contact is a stronger predictor of psychological adaptation than intergroup contact in the postcolonial environments.

## Conclusion

The current study aims to broaden the scope of adaptation research by exploring its applicability in a postcolonial context. The study has been crafted to develop a comprehensive predictive model for the psychological adaptation of Macau sojourners as they transition to universities in Mainland China, basing on two distinct groups of variables—identity-related variables, and intergroup-related variables. In summary, the study revealed that antecedents of successful cross-cultural adaptation drawn from the acculturation research, i.e., identity and quality of contact, continue to make significant contributions to psychological adaptation in the postcolonial context. However, the highlighted predictors of language proficiency and intergroup contact in inter-cultural adaptation are unrelated to psychological adaptation in intra-cultural adaptation of Macau students. Hence, Macau serves as an illustrative example of how tensions between local and national identities manifest in psychological adaptation within the postcolonial context. The current study also transported hypotheses and findings from inter-cultural settings and tested their validity and applicability in intra-cultural settings (Berry and Dasen, 1974; Berry et al., 1999).

Notably, findings of this research suggested that the variable of quality of contact is much more powerful and effective than the variable of intergroup contact in the Macau postcolonial context. This provided valuable implications for the Chinese government to improve educational sojourners’ psychological functioning in the postcolonial context. Based on the findings, one strategy that needs to be particularly highlighted is facilitating the optimal contact conditions between Macau students and the mainland students. Therefore, this study advocated Chinese higher education institutions should continue to implement integration policies, e.g., Macau students and the mainland students living and studying together, and at the same time, to facilitate more satisfying interactions between the two groups, e.g., Macau students and the mainland students sharing one academic criterion and having equal access to scholarships and other resources.

The findings also provided valuable implications for education institutions and Chinese policy makers. As demonstrated, psychological adaptation can pose great challenges for students relocating to their home country from a previously colonized area. Consequently, mainland universities and relevant supporting offices, such as the Student Affairs Office and the Office of Taiwan, Hong Kong, and Macau Affairs, should regularly organize intercultural communication workshops focusing on the socio-cultural and political disparities between Macau and the Mainland. These workshops should involve participation from both Macau and Mainland students. Moreover, Macau students and other stakeholders who are involved in these students’ experiences studying in Mainland China can use the time prior to college study to produce better outcomes in terms of their adaptation. It is not sufficient merely to organize intercultural communication workshops in universities; more importantly, the Macau high schools should incorporate the top-level design of intercultural intelligence into the school’s teaching, discipline construction, student management, and other aspects. Moreover, the study discovered that strengthening the Mandarin language skills of Macao students remains an important task. Although some colleges and universities in the Mainland arrange foundational Mandarin courses for students who have not achieved satisfactory results in the Joint Examination of Overseas Chinese and Students from Macao, the content of the courses is mostly about academic guidance for students to meet credit requirement; no courses concerning daily communication and intracultural differences are offered. Therefore, to help Macao students better adapt to the Mandarin-speaking environment, Mainland universities should offer introductory as well as advanced Mandarin courses with more in-class speaking practices or activities for all enrolled Macao students, especially for undergraduate students. The findings of the study also showed that identification with a Chinese national identity is highly important for good psychological states. Therefore, Chinese policy-makers should prioritize agendas for decolonization and renationalization of Macau students instead of strengthening a local identity. Overall, given the increasing number of Macau students studying in Mainland China, this research offers a timely exploration of their psychological adaptation.

## Limitations and future directions

The findings should be approached with caution, and it’s important to acknowledge several limitations. First, as the study specifically targeted university students, the results may not be applicable to other sojourners or expatriates. Second, the study, like other adaptation research, might be susceptible to self-selection bias in the sample: there is a possibility that Macau students who are more proficient in Mandarin and identify more strongly as Chinese than Macanese might be more inclined to pursue studies in Mainland China. Lastly, it’s important to note that, due to the cross-sectional design of this study, causality cannot be definitively established. Future studies may extend the current work by applying longitudinal or experimental designs. Also, it is recommended that this predictive model be investigated in the Hong Kong postcolonial context to further establish validity.

## Data availability statement

The raw data supporting the conclusions of this article will be made available by the authors, without undue reservation.

## Ethics statement

The studies involving humans were approved by the Ethics Committees of Xiamen University and Tsinghua University. The studies were conducted in accordance with the local legislation and institutional requirements. The participants provided their written informed consent to participate in this study.

## Author contributions

HF: Conceptualization, Data curation, Methodology, Formal analysis, Funding acquisition, Investigation, Supervision, Writing – original draft, Writing – review & editing. LZ: Data curation, Methodology, Funding acquisition, Writing – review & editing.

## Footnotes

^1^Retrieved on March 6, 2024, http://www.moe.gov.cn/fbh/live/2022/54857/mtbd/202209/t20220920_663330.html.

^2^The often-used geographical dividing line between northern and southern China is the Qinling–Huaihe Line corresponding roughly to the 33rd parallel.
